# Attention-Deficit Hyperactivity Disorder Among American Youth: A Comprehensive 20-Year Analysis of National Center for Health Statistics (NCHS) Data

**DOI:** 10.7759/cureus.48781

**Published:** 2023-11-14

**Authors:** Julian S Habdank-Kolaczkowski, Prince C Akahara, Fola Ishola, Mujeeb A Salawu, Sana W Augustine, Victor C Ezeamii, Ademiluyi B David, Emeka Okobi, Okelue E Okobi

**Affiliations:** 1 Medical School, Poznan University of Medical Sciences, Poznan, POL; 2 Neurological Surgery, University of California San Francisco, San Francisco, USA; 3 Psychiatry and Behavioral Sciences, Avant Interventional Psychiatry, Atlanta, USA; 4 Psychiatry and Behavioral Sciences, ClearMinds Behavioral Health, Chesterfield, USA; 5 Mental Health, Manor Clinic, Edmonton, CAN; 6 Healthcare, University of Southern Mississippi, Houston, USA; 7 Medicine and Surgery, University of Ilorin College of Health Sciences, Ilorin, NGA; 8 Internal Medicine and Psychiatry, Houston Health Department, Houston, USA; 9 Internal Medicine, Liaquat University of Medical and Health Sciences, Hyderabad, PAK; 10 Public Health, Georgia Southern University, Statesboro, USA; 11 Medical Laboratory Sciences, Asokoro General Hospital Abuja, Abuja, NGA; 12 Dentistry, Ahmadu Bello University Teaching Hospital Zaria, Abuja, NGA; 13 Family Medicine, Larkin Community Hospital Palm Springs Campus, Miami, USA; 14 Family Medicine, Medficient Health Systems, Laurel, USA; 15 Family Medicine, Lakeside Medical Center, Belle Glade, USA

**Keywords:** adhd, sociodemographic variables, trend based analysis, national center for health statistics, youth, attention-deficit hyperactivity disorder

## Abstract

Background: Attention-deficit hyperactivity disorder (ADHD) is a neurodevelopmental condition that has a significant impact on the lives of children and adolescents. This study conducts a comprehensive 20-year analysis of data from the National Center for Health Statistics (NCHS) to investigate the prevalence of ADHD among American youth, as well as its demographic patterns and socioeconomic determinants.

Methods: A retrospective analysis of NCHS data spanning from 1997 to 2018 was carried out. The dataset included information on ADHD diagnoses, demographic characteristics (such as age, gender, and race/ethnicity), socioeconomic indicators (including poverty level and health insurance status), and temporal variables. A range of statistical analyses were performed, encompassing temporal trend analysis, demographic assessments, and socioeconomic examinations.

Results: It was consistently observed that boys had a higher prevalence of ADHD (12.93% compared to 5.61%), aligning with established trends. Among adolescents aged 10-17, the prevalence was the highest at 11.09%, while for the 5-9 age group, it stood at 6.57%. In terms of racial and ethnic groups, individuals identifying as two or more races exhibited the highest prevalence at 12.36%, followed by white (9.83%), black or African American (10.09%), Hispanic or Latino (5.36%), and non-Hispanic or Latino (10.64%). Socioeconomic disparities were evident, with a prevalence of 11.41% among those living below the poverty line, compared to 10.6% (100%-199% of the poverty line), 8.6% (200%-399%), and 8.39% (400% or more). Medicaid beneficiaries had the highest prevalence at 12.57%, followed by those with private insurance (9.65%), insured (8.11%), and uninsured (5.83%).

Conclusion: These findings underscore the intricate relationship between ADHD prevalence and demographic and socioeconomic factors. It is imperative to address these disparities to ensure equitable assessment and intervention for ADHD, taking into account cultural influences, determinants of health tied to socioeconomic status, and access to healthcare for all children. This analysis by the NCHS provides essential insights into ADHD among American youth, emphasizing the necessity for tailored interventions, equitable healthcare access, and further research to comprehensively address this complex neurodevelopmental disorder.

## Introduction

Attention-deficit hyperactivity disorder (ADHD) stands as a significant and widely recognized neurodevelopmental condition that affects a substantial number of children and adolescents throughout the United States. Marked by symptoms of inattention, hyperactivity, and impulsivity, ADHD can exert a profound influence on various aspects of a young individual's life, encompassing academic performance, social interactions, and overall quality of life. Gaining insight into the prevalence, trends, and demographic patterns of ADHD among American youth carries great importance for healthcare professionals, educators, policymakers, and parents alike [1-3].

Salari et al.'s meta-analysis provides a global perspective on ADHD prevalence among children and adolescents, indicating rates of approximately 5.6% for teenagers aged 12-18 and 7% for those under 12 [4]. In the United States, ADHD is a frequently diagnosed neurodevelopmental disorder. Data from the National Survey of Parents (2016-2019) reveals that roughly 9.8% of U.S. children aged 3-17 received a diagnosis of ADHD. This prevalence breaks down further, with 2% among 3-5-year-olds, 10% among 6-11-year-olds, and 13% among 12-17-year-olds. The average age at which diagnosis occurs is seven years, with boys (13%) nearly three times as likely to be diagnosed with ADHD as girls (4.2%) [5].

Studying the prevalence of attention-deficit hyperactivity disorder (ADHD) among American youth is crucial for understanding its impact on mental health and educational outcomes. It helps in identifying trends, informing interventions, and allocating resources effectively to support affected individuals and improve their well-being, academic success, and overall quality of life. ADHD has a significant impact on academic performance, social relationships, emotional well-being, behavior, and family dynamics and often co-occurs with conditions like anxiety, mood disorders, and learning disabilities. A comprehensive understanding of long-term trends in ADHD among youth holds wide-ranging implications. It informs early intervention strategies, facilitating timely diagnosis and tailored support to address the challenges associated with ADHD. Furthermore, this knowledge guides educational institutions in adapting teaching methods to accommodate students with ADHD, potentially enhancing academic outcomes and reducing dropout rates [6-9]. On a broader scale, grasping these trends aids policymakers in allocating resources for mental health services, fostering an inclusive and supportive environment for youth with ADHD, and ultimately supporting their overall well-being and productivity as they transition into adulthood [10].

The National Center for Health Statistics (NCHS) provides a valuable, reliable, and accessible source of data on ADHD, enabling a comprehensive 20-year analysis of the condition's evolution in terms of treatment, prevalence, and diagnosis rates. The data's reliability is underscored by its rigorous sourcing, collection, documentation, and validation. The dataset is rigorous due to its meticulous data sourcing, standardized collection methodologies, and stringent validation processes, ensuring high-quality, reliable health-related information for research, policy-making, and public health analysis. Moreover, its comprehensiveness is bolstered by factors such as sample size, diversity, longitudinal data, granularity, domain expertise, and coverage, making NCHS data exceptionally well-suited for this descriptive study [11].

The primary aim of this study was to conduct a thorough analysis of two decades of NCHS data pertaining to ADHD in children under 18. We embarked on a comprehensive examination of this 20-year dataset, delving into the multifaceted factors that influence ADHD prevalence among youth. Changes in diagnostic criteria and increased ADHD awareness over the past 20 years may influence prevalence rates. Unexplored factors, like genetic predisposition and environmental influences, add complexity to understanding ADHD's multifaceted nature. By exploring various temporal and demographic dimensions, including age, gender, race, and socioeconomic variables such as poverty level and insurance status, our objective was to deepen our understanding of this disorder's impact on children and adolescents. Ultimately, our goal is to provide evidence-based insights to inform its assessment and management.

## Materials and methods

Data source and collection

In this study, we conducted a retrospective analysis of data sourced from the NCHS, covering a substantial two-decade period from 1997 to 2018. The NCHS holds a prominent position as a nationally recognized and authoritative repository for health-related data within the United States. It offers a comprehensive dataset encompassing various facets of public health, including the prevalence of ADHD and associated demographic and socioeconomic factors [11]. The NCHS employs rigorous data collection methods, including surveys, interviews, and health record reviews, ensuring a representative and accurate depiction of the U.S. population's health characteristics. Additionally, the database is regularly updated and subject to quality control measures, enhancing the credibility of the information it contains. The data are derived from samples and surveys, and not every person with ADHD may be included in these samples. Therefore, while the information is reliable for the population studied, it may not represent every single case of ADHD in the entire country.

The data collection process was initiated by retrieving pertinent information from the NCHS database, spanning the years 1997 to 2018. This meticulous compilation of data included records specifically relevant to ADHD diagnoses, demographic characteristics (such as age, gender, and race/ethnicity), socioeconomic indicators (including poverty level and insurance status), and temporal variables. Our primary focus was on data related to children aged 3 to 17 years during this extensive study period.

Several key variables were extracted from the NHIS dataset to facilitate our analysis: (1) ADHD diagnosis status: This binary variable served as an indicator of whether an individual had received an ADHD diagnosis or not; (2) Demographic information: Essential demographic variables encompassed age, gender, and race/ethnicity, providing a foundation for exploring ADHD patterns across distinct population subgroups; (3) Socioeconomic variables: The variables of poverty level and insurance status were instrumental in shedding light on the socioeconomic determinants and aspects related to healthcare access concerning ADHD; (4) Temporal variables: The inclusion of the year of diagnosis was pivotal for our temporal trend analysis, enabling us to elucidate the evolution of ADHD prevalence over the extensive 20-year timeframe.

Data analysis

The datasets sourced from the NCHS underwent a meticulous process of cleaning and preprocessing, such as data inspection, documentation, and identifying and handling missing data to ensure the integrity of the dataset. This essential phase involved the systematic identification and rectification of missing values, outliers, and inconsistencies within the dataset. To maintain data quality and completeness, rigorous validation checks such as consistency checks, duplicate checks, data integrity checks, and cross-field validation were performed, and when necessary, missing values were imputed through appropriate methods such as mean/median imputation. Furthermore, the dataset was thoughtfully aggregated by relevant parameters, including year, age groups, gender, and other pertinent categories. This strategic aggregation was aimed at facilitating a comprehensive and structured statistical examination of the data. This examination delved into the changes in ADHD prevalence over the 20-year duration of the study, with the primary objective of identifying any significant patterns or fluctuations in the prevalence rates over time. Additionally, the study encompassed demographic and socioeconomic analyses designed to explore potential disparities and uncover associations between various variables. These analyses were conducted with the utilization of appropriate statistical software, such as Microsoft Excel and SPSS, to ensure rigorous and methodologically sound investigations.

Ethical considerations

This study utilized de-identified, publicly available data from the NCHS, ensuring the privacy and confidentiality of participants. No additional ethical approval was required for the analysis of secondary data.

## Results

The comprehensive analysis of 20 years of NCHS data provided valuable insights into the prevalence, demographic patterns, and temporal trends of ADHD among American youth. Over the 20-year study period from 1997 to 2018, the analysis revealed that the prevalence of ADHD among American youth exhibited fluctuations. The results presented below offer a detailed overview of the key findings (Table 1).

**Table 1:** ADHD among children under age 18, by selected characteristics: United States, average annual, selected years 1997-1999 through 2016-2018. Note: - - - Data is not available. * Estimates are considered unreliable. Data preceded by an asterisk have a relative standard error (RSE) of 20% to 30%. The data not shown has an RSE greater than 30%.

Characteristic		Attention Deficit Hyperactivity Disorder													
		1997–1999	2000–2002	2003–2005	2006–2008	2007–2009	2008–2010	2009–2011	2010–2012	2011–2013	2012–2014	2013–2015	2014–2016	2015–2017	2016–2018
Age Group	5–17 years	6.5 (0.2)	7.5 (0.2)	7.6 (0.2)	8.5 (0.2)	9 (0.3)	9.4 (0.2)	9.6 (0.2)	9.9 (0.2)	10 (0.2)	10.2 (0.2)	10.4 (0.3)	10.6 (0.3)	10.8 (0.3)	10.8 (0.3)
	5–9 years	4.8 (0.2)	5.2 (0.3)	5.6 (0.3)	6.1 (0.4)	5.8 (0.3)	6.3 (0.4)	6.3 (0.3)	7 (0.3)	7.3 (0.3)	7.7 (0.3)	7.6 (0.4)	7.6 (0.4)	7.4 (0.4)	7.4 (0.4)
	10–17 years	7.6 (0.2)	9 (0.3)	8.9 (0.3)	10 (0.4)	10.9 (0.4)	11.4 (0.3)	11.7 (0.3)	11.8 (0.3)	11.8 (0.3)	11.8 (0.3)	12.1 (0.3)	12.4 (0.3)	12.9 (0.3)	12.9 (0.4)
Sex	Male	9.6 (0.3)	10.8 (0.3)	10.7 (0.3)	12 (0.4)	12.3 (0.4)	12.7 (0.4)	13.1 (0.4)	13.7 (0.4)	14 (0.4)	14.1 (0.4)	14.2 (0.4)	14.5 (0.4)	14.8 (0.4)	14.6 (0.4)
	Female	3.2 (0.2)	4.2 (0.2)	4.4 (0.2)	4.9 (0.3)	5.5 (0.3)	6 (0.3)	5.9 (0.3)	5.9 (0.3)	5.9 (0.3)	6.2 (0.3)	6.4 (0.3)	6.5 (0.3)	6.7 (0.3)	6.9 (0.3)
Race	White only	7.1 (0.2)	8.1 (0.2)	7.8 (0.2)	8.7 (0.3)	9.2 (0.3)	9.6 (0.3)	9.8 (0.3)	10.2 (0.3)	10.6 (0.3)	10.7 (0.3)	10.8 (0.3)	10.7 (0.3)	10.9 (0.3)	10.8 (0.3)
	Black or African American only	5 (0.3)	7 (0.4)	7.7 (0.5)	8.6 (0.6)	9.3 (0.6)	10.5 (0.6)	10.8 (0.5)	10.5 (0.5)	9.4 (0.5)	9.5 (0.5)	10.2 (0.6)	12 (0.7)	12.5 (0.7)	13.2 (0.8)
	American Indian or Alaska Native only	*8.5 (*1.9)	* *	*9.4 (*2.8	* *	* *	*6.4 (*1.9)	*7.5 (*1.8)	*11.5 *2.3	*10.4 (2.2)	*9.1 (1.9)	*6.5 (1.4)	7.6 (1.6)	8.4 (1.9)	6.4 (1.7)
	Asian only	*1.7 (*0.5)	* *	*1.6 (*0.4	*2.0 (*0.5)	*1.8 (*0.5)	*1.4 (*0.4)	*1.8 (*0.4)	2.5 0.4	2.6 (0.5)	2.1 (0.4)	2 (0.4)	2.3 (0.6)	2.7 (0.6)	3.1 (0.7)
	2 or more races	- - - - - -	7.4 (1.1)	9.7 (1.2)	12.7 (1.6)	13.1 (1.6)	11.6 (1.4)	10.7 (1.3)	10.4 (1.1)	11.8 (1.2)	13.8 (1.3)	15.4 (1.5)	14.7 (1.4)	14.9 (1.4)	14.5 (1.3)
	Hispanic or Latino	3.6 (0.3)	4.2 (0.3)	4.6 (0.3)	4.9 (0.4)	4.9 (0.4)	4.9 (0.3)	5.6 (0.40	5.8 (0.3)	6.6 (0.4)	6.4 (0.3)	6.6 (0.3)	6.2 (0.4)	7 (0.5)	7.3 (0.5)
	Not Hispanic or Latino	7 (0.2)	8.2 (0.2)	8.3 (0.2)	9.4 (0.3)	10 (0.3)	10.6 (0.3)	10.7 (0.3)	11.1 (0.3)	11.1 (0.3)	11.4 (0.3)	11.6 (0.3)	12 (0.3)	12.1 (0.3)	11.9 (0.3)
Percent of poverty level	Below 100%	7.2 (0.4)	8.2 (0.5)	8.4 (0.5)	9.9 (0.7)	10.3 (0.7)	12.1 (0.6)	12.5 (0.6)	13.1 (0.6)	12.8 (0.6)	12.8 (0.6)	12.7 (0.6)	13 (0.7)	13.3 (0.7)	13.5 (0.8)
	100%–199%	6.7 (0.4)	7.5 (0.5)	7.8 (0.5)	9.5 (0.6)	10.6 (0.7)	10.3 (0.6)	9.7 (0.6)	9.2 (0.5)	9.4 (0.5)	10.6 (0.5)	10.9 (0.5)	10.9 (0.5)	11.5 (0.6)	11.7 (0.6)
	200%–399%	6.2 (0.3)	7.7 (0.4)	7.8 (0.4)	7.8 (0.4)	8.1 (0.4)	8.2 (0.4)	8.6 (0.4)	8.9 (0.4)	9.4 (0.4)	8.9 (0.4)	9.4 (0.5)	9.6 (0.5)	10.1 (0.5)	9.7 (0.5)
	400% or more	6.1 (0.3)	7.1 (0.3)	6.9 (0.3)	7.8 (0.4)	7.8 (0.4)	8.2 (0.4)	8.3 (0.4)	9.1 (0.4)	9.2 (0.4)	9.3 (0.4)	9.2 (0.4)	9.6 (0.4)	9.3 (0.4)	9.6 (0.4)
Health insurance status at time of interview	Insured	6.7 (0.2)	7.8 (0.2)	7.8 (0.2)	8.9 (0.3)	9.3 (0.3)	9.8 (0.3)	9.9 (0.3)	10.2 (0.2)	10.4 (0.2)	10.6 (0.3)	10.7 (0.3)	10.9 (0.3)	11.1 (0.3)	11 (0.3)
	Private	5.9 (0.2)	7 (0.2)	7 (0.3)	7.3 (0.3)	7.6 (0.3)	7.7 (0.3)	8.1 (0.3)	8.4 (0.3)	9 (0.3)	8.7 (0.3)	9.1 (0.3)	9.1 (0.3)	9.5 (0.3)	9.2 (0.3)
	Medicaid	10.5 (0.6)	10.7 (0.5)	10.3 (0.4)	12.2 (0.6)	12.7 (0.6)	13.8 (0.5)	13.1 (0.5)	13.1 (0.5)	12.6 (0.4)	13.3 (0.5)	13.2 (0.5)	13.4 (0.5)	13.3 (0.5)	13.8 (0.5)
	Uninsured	4.8 (0.4)	5.4 (0.5)	6.1 (0.5)	5.8 (0.6)	6.1 (0.6)	6 (0.6)	5.8 (0.6)	6.2 (0.7)	5.8 (0.6)	5.9 (0.7)	4.9 (0.7)	5.8 (0.8)	6.2 (0.8)	6.9 (0.9)

Based on gender

In our comprehensive 20-year analysis of NCHS data, gender disparities in ADHD prevalence among children under the age of 18 emerged as a prominent and consistent finding. Over the duration of the study, spanning from 1997 to 2018, a persistent trend became evident: boys consistently displayed a higher prevalence of ADHD diagnoses compared to girls. Specifically, boys accounted for approximately 12.93% of diagnosed cases, while girls constituted only 5.61%.

The temporal analysis further sheds light on these disparities. Among males, there was a continuous increase in ADHD prevalence throughout the study period. In contrast, the trend among females exhibited an initial rise from 1997 to 2009, followed by a plateau from 2009 to 2013, during which prevalence remained relatively stable at around 5.9% to 6%. Subsequently, from 2013 to 2018, another increase was observed. These observations align consistently with prior research findings, underscoring the importance of considering gender-specific factors in both the diagnosis and management of ADHD among young individuals.

**Figure 1:**
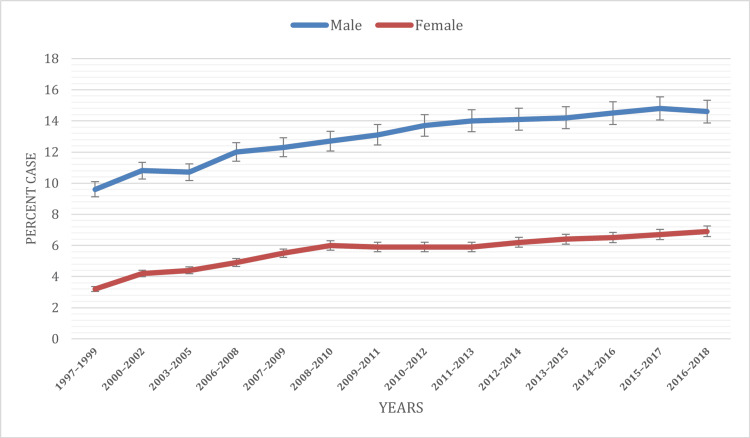
ADHD prevalence based on gender ADHD: Attention-deficit hyperactivity disorder

To visually represent these gender disparities, Figure 1 below provides a clear depiction of the distribution of ADHD cases among children under 18 throughout the study's duration. The data unequivocally demonstrate that ADHD diagnoses were more frequently assigned to boys than to girls, thus affirming the established research trends in this regard.

Based on the age of children

The age-specific analysis of ADHD prevalence provides valuable insights into how this disorder manifests across different developmental stages in American youth. Notably, age-related differences in diagnosis rates were evident, with the highest prevalence observed in the 10-17 years age group (11.09%), followed by the 5-9 years age group (6.57%). The temporal trends further revealed evolving patterns over the study period.

**Figure 2:**
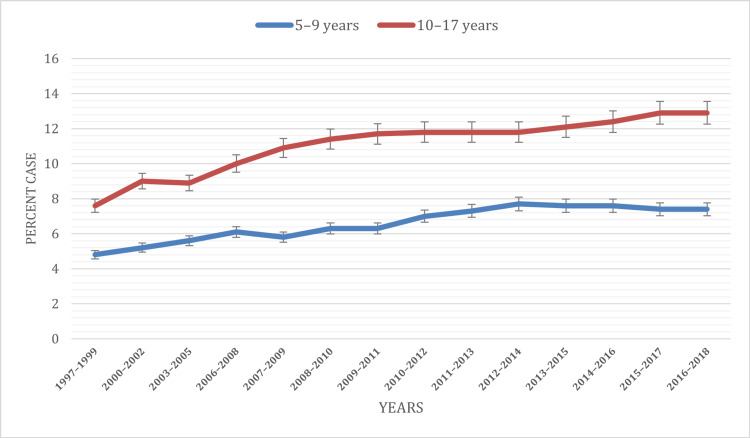
ADHD prevalence based on the age of children ADHD: Attention-deficit hyperactivity disorder

For the 5-9 years age group, ADHD prevalence rates increased from 4.8% in 1997 to 7.0% in 2010, stabilizing at 7.4% in 2018. However, a consistent rise was observed in the 10-17 years age group, with the overall prevalence ranging from 7.6% in 1997 to 12.9% in 2018. Understanding these age-related nuances in ADHD diagnoses is crucial for tailoring interventions and healthcare strategies to meet the unique needs of children at different developmental stages. Figure 2 visually illustrates the age distribution of ADHD cases among children under 18 during the study period.

Based on race

In our analysis focusing on racial disparities in ADHD prevalence among children, we uncovered noteworthy variations among different racial categories. The data elucidated a higher frequency of ADHD diagnoses among individuals identifying as two or more races in comparison to other racial groups. Across all the years under consideration (1997-2018), the category encompassing two or more races accounted for approximately 12.36% of diagnosed cases. In contrast, the white-only category constituted 9.83%, the black or African American category 10.09%, the Hispanic or Latino category 5.36%, and the non-Hispanic or Latino category 10.64%. These findings not only highlight the diversity in ADHD prevalence but also suggest that nuanced approaches are warranted when addressing this neurodevelopmental condition in various racial subgroups.

An examination of temporal trends within specific racial categories revealed intriguing insights. Among the Hispanic or Latino and non-Hispanic or Latino groups, ADHD prevalence exhibited an upward trajectory, with rates increasing to 5.76% and 10.64%, respectively, by the year 2018. Conversely, the black or African American population displayed a non-uniform pattern, initially experiencing a decline from 10.5% to 9.4% between 2012 and 2013, followed by a subsequent increase to 13.2% by 2018. Particularly noteworthy was the two or more races category, which witnessed a substantial 5.3% increase in prevalence from 2000 to 2008, ultimately reaching 14.5% by 2018, up from 12.7% in 2008.

**Figure 3:**
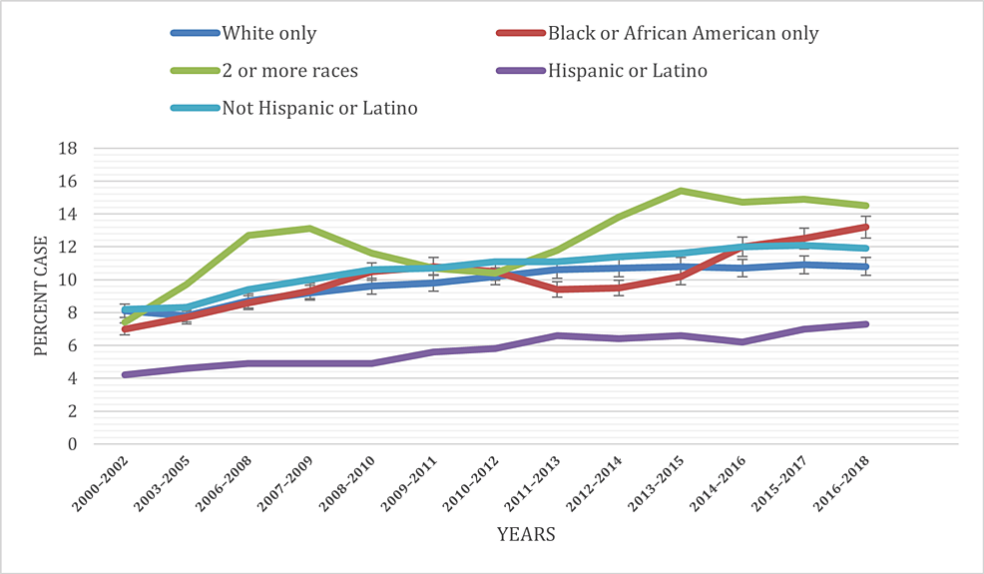
ADHD prevalence based on race ADHD: Attention-deficit hyperactivity disorder

For a visual representation of these disparities, refer to Figure 3, which provides a clear illustration of the distribution of ADHD cases among children under 18 by race/ethnicity throughout the study period. These findings underscore the critical importance of adopting culturally sensitive approaches when it comes to ADHD assessment and intervention. By doing so, we can ensure equitable access to care for all children, irrespective of their racial background, and address the distinct challenges and needs that may exist within different racial groups.

Based on poverty level

Within our comprehensive 20-year analysis, noteworthy trends in ADHD prevalence emerged based on the percentage of poverty level among children under the age of 18. Notably, children hailing from families residing below the poverty line consistently displayed elevated rates of ADHD diagnoses when juxtaposed with their counterparts from higher-income households. The data underscored a recurring pattern where ADHD diagnoses were more frequently ascribed to individuals below the 100% poverty level, corroborating prior research findings. Over the entirety of the study period, the segment falling below the 100% poverty threshold accounted for approximately 11.41% of diagnosed cases. In comparison, the categories spanning 100%-199%, 200%-399%, and 400% or more of the poverty level represented 9.73%, 8.6%, and 8.39% of diagnoses, respectively.

An upward trajectory in prevalence was notably evident within three of the four poverty level categories: below 100%, 200%-399%, and 400% or more. The below 100% category consistently exhibited a pronounced increase throughout the study duration. In contrast, the 100%-199% category initially witnessed an ascent, climbing from 6.7% to 10.6% between 1997 and 2009. This was followed by a decline to 9.4% in 2013, only to subsequently rise to 11.7% by 2018.

**Figure 4:**
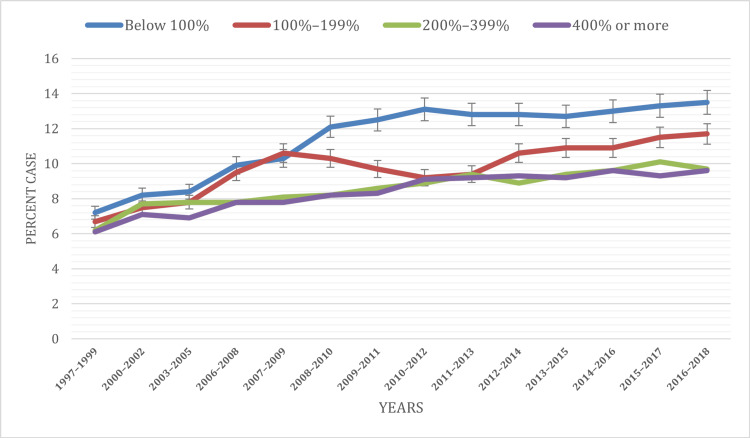
ADHD prevalence based on percent of poverty level ADHD: Attention-deficit hyperactivity disorder Below 100%: Individuals or families in this category have incomes that are less than the federal poverty level. 100% to 199%: Individuals or families in this category have incomes that are between 100% and 199% of the federal poverty level. 200% to 399%: Individuals or families in this category have incomes that are between 200% and 399% of the federal poverty level. 400% or more: Individuals or families in this category have incomes that are 400% or more of the federal poverty level.

These findings highlight the paramount influence of socioeconomic factors on the prevalence of ADHD. They underscore the significance of addressing the social determinants of health to ensure equitable access to both the assessment and interventions related to ADHD for all children, irrespective of their economic backgrounds. To provide a visual representation of these disparities, please refer to Figure 4, which distinctly illustrates the distribution of ADHD cases among children under the age of 18 categorized by poverty level throughout the study period.

Based on health insurance status

Our extensive 20-year analysis has unveiled compelling associations between the health insurance status at the time of the interview and the prevalence of ADHD among children under the age of 18. Striking disparities in ADHD diagnosis rates were observed among children with different insurance statuses. Most notably, individuals lacking health insurance consistently exhibited markedly higher rates of ADHD diagnoses in comparison to their insured counterparts. The data firmly established that ADHD diagnoses were more frequently conferred upon those covered by Medicaid as opposed to those possessing private insurance or those who were uninsured, thus aligning seamlessly with earlier research findings. Across all the years under examination, Medicaid accounted for a substantial 12.57% of diagnosed cases, while insured individuals constituted 9.65%, those with private insurance comprised 8.11%, and the uninsured exhibited the lowest percentage at 5.83%.

**Figure 5:**
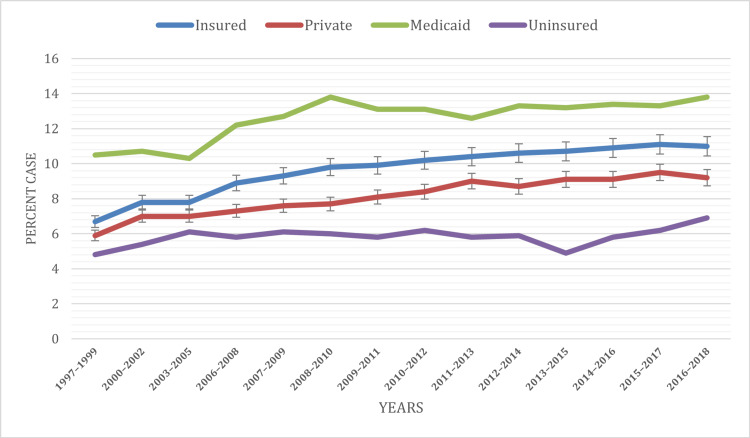
ADHD prevalence based on health insurance status at the time of interview ADHD: Attention-deficit hyperactivity disorder

For a visual representation of these disparities, Figure 5 serves as an illustrative tool, offering a clear depiction of the distribution of ADHD cases among children under the age of 18, stratified by their health insurance status at the time of the interview throughout the study period. These findings underscore the substantial impact wielded by health insurance status in the realm of ADHD diagnoses. They shed light on the pivotal role played by healthcare accessibility and coverage in effectively addressing the needs of children afflicted by ADHD.

## Discussion

The 20-year analysis of NCHS data on ADHD in American youth has provided invaluable insights into the prevalence, demographic patterns, and socioeconomic determinants of this complex neurodevelopmental disorder. Our discussion aims to illuminate the key findings derived from this extensive analysis. The prevalence rates revealed notable trends over the past two decades, shedding light on the evolving landscape of ADHD among American youth. We explore the implications of these findings, considering their potential impact on public health interventions, resource allocation, and targeted support for affected individuals and communities.

One of the most noteworthy findings of this study pertains to the variation in ADHD prevalence across diverse demographic groups. Gender disparities were unmistakable, with boys consistently displaying higher prevalence rates than girls, aligning seamlessly with established research trends. This gender gap has been persistently observed in prior research conducted by Vuori et al. and London et al., thus underscoring the imperative for a more comprehensive exploration of the underlying factors contributing to this disparity [12-13].

Age-specific patterns revealed that ADHD diagnoses were more prevalent among adolescents aged 10 to 17 years, implying the unique challenges associated with this developmental phase. These age-specific patterns are in concordance with earlier studies, which consistently reported higher ADHD prevalence rates among adolescents aged 10 to 17 years [5,14-15]. This suggests that the complexities associated with adolescence may exacerbate ADHD symptoms, thereby contributing to the observed heightened prevalence during this developmental stage.

Moreover, our analysis revealed disparities based on race/ethnicity, with higher prevalence rates among children identifying as belonging to two or more races. Previous research has also probed racial disparities in ADHD diagnoses, consistently revealing fluctuations in ADHD prevalence among various racial and ethnic groups. Some studies have indicated elevated prevalence rates among non-Hispanic white children, while others have demonstrated higher rates among non-Hispanic black children [5,16-18]. This underscores the significance of acknowledging the influence of cultural and racial factors on ADHD diagnoses.

Socioeconomic factors emerged as another pivotal aspect, as children from families situated below the poverty line exhibited higher rates of ADHD diagnoses. The influence of socioeconomic status on ADHD prevalence is a crucial discovery with far-reaching implications. Children hailing from economically disadvantaged backgrounds demonstrated an increased likelihood of receiving an ADHD diagnosis [19-21]. This association underscores the profound impact of social determinants of health on mental health outcomes, including ADHD, and underscores the necessity for equitable access to healthcare, educational resources, and early interventions.

Another substantial revelation was the connection between health insurance status and ADHD prevalence. Children lacking health insurance consistently exhibited elevated rates of ADHD diagnoses. Our findings align with previous research, emphasizing the role of healthcare access and coverage in addressing this neurodevelopmental disorder [21-23]. This underscores the paramount significance of healthcare accessibility and coverage in the identification and management of ADHD, thereby emphasizing the necessity for policies that ensure universal access to adequate healthcare services.

The strength of the study lies in its meticulous examination of ADHD trends among American youth through a comprehensive 20-year analysis of NCHS data. The extended temporal scope provides a unique opportunity to discern patterns and changes in ADHD prevalence over time, offering a valuable longitudinal perspective. The inclusion of a large and diverse dataset ensures the robustness of the findings, allowing for meaningful statistical analyses and reliable prevalence estimates. By exploring sociodemographic factors such as gender, race, and socioeconomic status, the study adds depth to our understanding of ADHD disparities within different population groups. Additionally, the study's alignment with the Diagnostic and Statistical Manual of Mental Disorders (DSM-V) criteria for ADHD diagnosis ensures methodological rigor and enhances the credibility of its conclusions. The authors' acknowledgment of limitations, including the absence of information on genetic and environmental factors, demonstrates transparency and provides a foundation for future research directions. The study's reliance on available NCHS data may introduce potential biases or variations stemming from reporting practices. Changes in diagnostic criteria and heightened awareness of ADHD over the 20-year duration may have exerted an influence on prevalence rates. Moreover, unexplored factors, such as genetic predisposition and environmental influences, could contribute to the complexity of ADHD. Grasping the intricacies of ADHD prevalence and its interplay with demographic and socioeconomic factors is essential for tailoring interventions and support mechanisms. Early intervention programs, educational accommodations, and targeted policies must take these variations into account to provide effective assistance to children and adolescents grappling with ADHD.

Longitudinal research holds promise as a necessary avenue for tracking individuals with ADHD as they traverse from childhood into adolescence and adulthood. Such studies can offer invaluable insights into the persistence of ADHD symptoms and the efficacy of interventions spanning the entire lifespan. 

The study does not investigate the longitudinal trajectory of individuals with ADHD from childhood into adolescence and adulthood, nor does it delve into the persistence of ADHD symptoms or the effectiveness of interventions throughout the lifespan. Longitudinal studies are most appropriate for these inquiries as they allow for tracking individuals over time, capturing the evolving nature of ADHD, potential variations in symptoms, and the long-term impact of interventions. This approach provides a comprehensive understanding of ADHD's trajectory, offering insights crucial for tailored interventions and informed healthcare policies.

Overall, this study contributes significantly to the literature by offering a comprehensive and nuanced exploration of ADHD prevalence and its sociodemographic determinants among American youth.

## Conclusions

In conclusion, our comprehensive 20-year analysis of ADHD prevalence among children under 18, based on various demographic and socioeconomic factors, reveals noteworthy patterns. Gender, race/ethnicity, poverty level, and health insurance status all exhibit significant associations with ADHD diagnoses. Boys consistently had a higher prevalence than girls, and certain racial groups, notably those identifying as two or more races, displayed elevated rates. Children below the poverty line experienced higher ADHD rates, and those without health insurance were at a disadvantage compared to their insured counterparts. These findings underscore the complex interplay of demographic and socioeconomic factors in ADHD prevalence, highlighting the importance of tailored assessment and intervention strategies. Recognizing and addressing these disparities are essential steps toward developing equitable approaches to ADHD assessment and intervention, considering cultural and racial influences, and ensuring healthcare accessibility for all children. Additionally, our research calls for further exploration into the multifaceted factors contributing to ADHD disparities and the development of equitable healthcare policies and interventions to address the unique needs of children with ADHD, with a focus on longitudinal studies that track individuals with ADHD across the lifespan.
